# Does Transcranial Direct Current Stimulation (tDCS) Improve Disgust Regulation Through Imagery Rescripting?

**DOI:** 10.3389/fnhum.2019.00192

**Published:** 2019-06-06

**Authors:** Jakob Fink, Cornelia Exner

**Affiliations:** Department of Clinical Psychology and Psychotherapy, University of Leipzig, Leipzig, Germany

**Keywords:** transcranial direct current stimulation, brain stimulation, disgust, imagery rescripting, emotion regulation, visual cortex, prefrontal cortex, neuropsychological mechanism

## Abstract

The first pilot studies have shown the potential of imagery rescripting (ImR) for reducing contamination-related pathological disgust, although the effects were rather small. The aim of the present study is to investigate whether the effects of ImR in reducing disgust can be further increased by transcranial direct current stimulation (tDCS). tDCS is a non-invasive method of brain stimulation that has been successfully used multiple times to support emotion-regulation strategies. In the present study, disgust was induced via images related to individualized sources of disgust. Fifty-eight healthy volunteers took part in two parallel experiments. The two groups were matched by age, highest educational level and gender, and were tested under two emotion-regulation conditions, namely an ImR condition and a control condition. Participants performed three trials on the first day and three trials on the second day. Across both days they performed three trials under each of the two emotion-regulation conditions in a randomized order. On one day active stimulation was applied, while on the other day participants were sham stimulated. The combination of emotion-regulation and stimulation condition was balanced across subjects. The only difference between the two groups was the localization of tDCS stimulation: one group was stimulated over the dorsolateral prefrontal cortex and the other group was stimulated over the visual cortex (VC). This experimental manipulation was implemented to gain further insights into the underlying neuropsychological mechanisms of imagery. ImR was conducted via a previously-recorded audio file. The results confirm the effect of ImR on the reduction of disgust. However, with the present experimental design we were not able to show that supplementary tDCS of the VC or the dorsolateral prefrontal cortex lead to improvement.

## Introduction

High disgust sensitivity seems to play a pathogenic role for the subtype of obsessive–compulsive disorder (OCD), whereby those afflicted predominantly show concerns about contamination and washing (C-OCD; Cisler et al., 2010). Challenging disgust experiences with cognitive behavioral therapy using exposure and response prevention (ERP) has proved more difficult than for other subtypes of OCD (ERP, Rosa-Alcazar et al., 2008). Disgust seems to result in slower habituation and stronger resistance to extinction compared to fear (Smits et al., 2002; Cougle et al., 2007; Olatunji et al., 2009; Mason and Richardson, 2010; Adams et al., 2011). One emotion regulation strategy, which might support ERP in changing pathological disgust, is imagery rescripting (ImR). ImR is supposed to change the affective meaning of aversive memories and intrusive images (Wild et al., 2008). Mental imagery is thought to be the mechanism behind ImR (Holmes and Mathews, 2005) and is defined as “representations and the accompanying sensory information without a direct external stimulus” (Pearson et al., 2015, p. 590). Even though treatment with ImR shows positive effects, particularly for PTSD and social anxiety (Morina et al., 2017), there is only one study so far, by our group, that has applied ImR in the context of C-OCD to change levels of disgust (Fink et al., 2018). In this laboratory study, we found that ImR and cognitive reappraisal were superior to control conditions (counting fishes) in changing levels of disgust for a clinical group of C-OC patients and a matched, healthy control group. Because the results of ImR in challenging disgust were promising, but only moderately significant, further ways to improve ImR are needed.

One way to reduce OCD symptoms and support emotion regulation strategies is transcranial direct current stimulation (tDCS), a “non-invasive” brain stimulation method with polarity-dependent effects on cortical excitability (Nitsche and Paulus, 2000). There are two ways to apply tDCS in targeting psychopathology: first, the direct approach by up or down stimulating disorder relevant hyperactive or hypoactive brain areas; second, by indirectly targeting psychopathology through stimulating brain areas which seem responsible for psychological compensation processes. The first way has been used by several studies in the context of OCD, and which are summed up in a recent review (Brunelin et al., 2018). The results show that there are moderate but promising effects of cathodal tDCS in reducing OCD symptoms for therapy-resistant patients. The cathodal electrode was mainly located over the pre-supplementary motor cortex (pre-SMA) or the lateral orbitofrontal cortex (OFC). Bais et al. (2014) proposed that cathodal stimulation on the OFC or pre-SMA might improve the OCD-impaired cortico-striato-thalamo-cortical circuitry. The reviewed studies applied tDCS in approximately 20 daily sessions for 20 min, with a direct current of 2–3 mA. Even though the first results seem promising in reducing OCD symptoms (Y-BOCS scores), these studies often lack sham-controlled conditions and only investigated small populations. Furthermore, relatively high current was applied, which increases the possibility of negative side effects and might therefore not be unproblematic (Davis, 2014). Although tDCS has been successfully applied repeatedly to support emotion regulation strategies (Boggio et al., 2009; Peña-Gómez et al., 2011; Feeser et al., 2014), the second way to indirectly reduce OCD symptoms by fostering psychological compensation processes has not yet been approached in the context of OCD. However, studies using tDCS in emotion regulation strategies did so successfully by applying smaller current (1–2 mA) within single sessions. Thus, supporting compensatory psychological processes with tDCS to reduce psychopathology might be a promising way to boost psychotherapeutic approaches but with smaller applied current and reduced risk of side effects.

In the present study, tDCS was applied to increase the effect of ImR on disgust regulation, thus aimed at fostering psychological compensation processes. Because malfunctioning regulation processes often are accompanied with extenuated neural activity (e.g., Feeser et al., 2014), an increase of brain activity in the related areas is necessary to improve compensation. This is why anodal tDCS was applied but not cathodal tDCS. There are several studies showing that the same neural regions are active during mental imagery as during actual perception (Kosslyn et al., 2001; see for review, Pearson et al., 2015). If greater vividness of the disgust-related mental images facilitates the process of changing the negative image to a positive one, then stimulating the visual cortex (VC) should result in better disgust reduction. If on the other hand ImR is primarily an active emotion regulation strategy in which top–down cognitive processes are involved, then stimulating the dlPFC might result in better disgust reduction. This is why the anodal electrodes were localized for one group on the VC and for the other group on the dlPFC.

An analog sample of healthy participants was studied. Due to the dimensional nature of OCD (Abramowitz et al., 2003), OCD-related deficits in compensation processes are also investigable in people with a tendency for OCD. The experimental ImR procedure of Fink et al. (2018) was adopted for the present study using 6-min, approved auditory instructions for guiding ImR. During the ImR task, participants were told how to change a disgust-inducing picture into a neutral or positive picture. To control for laboratory exposure and within session habituation, the authors included a non-intervention, control condition in which participants had to perform a counting task. In the experimental procedure, the same disgust-eliciting picture was presented and rated before and after the ImR procedure or the control task. Hypothesis 1a predicts that disgust reduction is significantly stronger through ImR compared to the control condition after active stimulation of the VC (group 1). In hypothesis 1b we expect that disgust reduction is significantly stronger through ImR compared to the control condition after active stimulation of the prefrontal cortex (group 2). An explorative hypothesis 2 was formulated that examined whether disgust reduction through ImR is more strongly enhanced after active tDCS of the dorsolateral prefrontal cortex or after active tDCS of the VC. Furthermore, the effects of washing symptom severity (C-OC symptoms), habitual use of imagery and habitual use of reappraisal and suppression on tDCS during imagery-based disgust reduction were investigated.

**Table 1:** Demographics and personal characteristics by group.

	Study 1: VC (*N* = 29)		Study 2: dlPFC (*N* = 29)		Statistical analysis		
	Mean	*SD*	Mean	*SD*	*t*	*p*-value	*d*
Age (years)	22.55	5.44	22.38	3.43	0.144	0.886	0.037
Gender (male:female; % male)	21:8	29%	21:8	29%	–	–	–
Padua IR, washing	6.34	4.71	6.45	4.7	−0.083	0.934	0.024
ERQ reappraisal	4.71	0.68	5.02	0.75	−1.64	0.108	0.433
ERQ suppression	2.9	1.04	3.09	0.79	−0.82	0.417	0.206
SUIS – 17 items	59.69	8.95	57.17	9.71	1.03	0.309	0.270

*VC, visual cortex; dlPFC, dorsolateral prefrontal cortex; Padua IR, Padua Inventory – Revised; ERQ, Emotion Regulation Questionnaire; SUIS, Spontaneous Use of Imagery Scale.*

## Materials and Methods

### Participants

We calculated the necessary sample size in line with our hypothesis with G^∗^power (V. 3.1; Faul et al., 2007) to be 26 per group assuming a medium effect size (*f* = 0.25*)* based on the findings of Fink et al. (2018), a power of 0.95 and an alpha of 0.05. Two consecutive studies were performed. Twenty-nine subjects participated in each of the two experiments. All participants were native German speakers. Participants were students from the University of Leipzig and received 25 Euro or 2.5 h student course credit. The absence of any current psychological disorder was confirmed with the *Mini-International Neuropsychiatric Interview* based on DSM-IV (M.I.N.I., Lecrubier et al., 1997; German version of Ackenheil et al., 1999). Exclusion criteria were ages under 18 or above 65, pregnancy, use of psychotropic drugs, addiction to psychotropic substances, current psychotherapeutic treatment, neurological diseases, metal implants in head or the upper part of the body and insufficient German skills. The first study group (stimulated over the VC) consisted of 21 females (72%) and 8 males (29%), and the average age was 22.55 years (*SD* = 5.44). The second study group (stimulated over the prefrontal cortex) also consisted of 29 participants, who were matched to the first group by age, gender and level of education. Therefore, this group also consisted of 21 females (72%) and 8 males (29%) and the average age was 22.38 years (*SD* = 3.43). Given that all participants in both groups were university students, this means that all had an *Abitur* (German school leaving exam) and therefore had completed German secondary schooling. The demographics and personal characteristics are displayed for each group in Table 1.

### Measures

The various subtypes of subclinical OCD symptoms were assessed by applying the four-point scaled Padua Inventory – Palatine Revision (PI-PR; German: Burns et al., 1996; Gönner et al., 2010), ranging from 0 (not at all) to 4 (very much). The measure assessed the OCD subtypes, contamination and washing (4 items), checking (6 items), numbers (3 items), dressing and grooming (3 items), rumination (3 items), and harmful obsessions and impulses (5 items). The PI-PR has been reported to have an acceptable internal consistency (Cronbach’s α > 0.78). The Spontaneous Use of Imagery Scale (SUIS; German: Reisberg et al., 2003; Nelis et al., 2014; Görgen et al., 2016) was used to assess the habitual use of imagery. The responses were given on a five-point Likert scale, ranging from 1 (always) to 5 (never). The German revision by Görgen et al. (2016) differs from the original SUIS by including six additional items and eliminating item 6. This version therefore results in a 17-item measure, which has been reported to have high internal consistency (Cronbach’s α > 0.85). To measure the habitual use of the emotion-regulation strategies cognitive reappraisal (six items) and expressive suppression (four items), the Emotion Regulation Questionnaire (ERQ; Gross and John, 2003; Abler and Kessler, 2009) was applied. The responses are given on a seven-point Likert scale, ranging from 1 (strongly disagree) to 7 (strongly agree). The ERQ has been reported to have acceptable internal consistency (Cronbach’s α > 0.74).^1^

### Experimental Design

The influence of tDCS during ImR on disgust reduction was tested separately for each of two locations in two 2 × 2 (2 emotion-regulation conditions × 2 tDCS conditions) within-subject designs. Each participant performed both of the experimental emotion-regulation conditions three times (trials) over 2 days, in randomized order (in total six trials). Furthermore, active and sham tDCS was also performed within-subjects, each on one of the 2 days, in randomized order. As this design resulted in an unequal number of emotion-regulation × stimulation conditions per subject, the combinations were balanced across participants (Appendix D). In order to answer the explorative hypothesis, the studies were compared using the effect sizes. The study was approved by the local ethics committee of the University of Leipzig (458-15-21122015). We have thoroughly reported sample size determination, data exclusion (if any), alterations and measures in the study.

### Stimuli and Material

The same 21 disgust-inducing pictures were used as emotional stimuli to induce emotional arousal with negative valence in this study as in the study of Fink et al. (2018). Of these, 16 pictures were selected from the Disgust-RelaTed-Images database (DIRTI, Haberkamp et al., 2017)^2^ and the other five pictures were selected from the internet. All pictures used in the present study had been previously validated by either Haberkamp et al. (2017) or Fink et al. (2018). The pictures were associated with seven categories of disgust. From this picture pool, six pictures were selected for each participant, which best matched the most likely source of disgust experience for that individual.

To individualize the disgust induction, the participants first answered 14 questions regarding general, disgust-related situations. The questionnaire was taken from the study of Fink et al. (2018). For each participant, the six pictures for the experiment were chosen from the two disgust categories that had elicited the highest scores for disgust experienced in the prior screening. Each disgust category contained three pictures and two corresponding questions for screening. The seven disgust categories were: (a) Bodily products (feces), (b) Bodily products (vomit), (c) lack of hygiene and presence of disease, (d) food, (e) insects, (f) garbage and mold, and (g) decomposing animal corpses. All the pictures were 16 cm wide and 12 cm long and were presented on a white screen, 30.5 cm wide and 13.5 cm long. The participants were seated about 50 cm from the front of the screen and responded using a keyboard. Psychtoolbox, based on MATLAB^©^ (Kleiner et al., 2007), was used to run the experiment on a ThinkPad laptop with a 14″ TFT monitor.

A direct current of 1 mA was generated by a battery-driven stimulator (neuroConn GmbH, Ilmenau, Germany) and continuously delivered via a pair of water-soaked sponge electrodes (with 25 cm^2^ surface area). At the end of stimulation, the current was reduced to 0 mA over 5 s. The electrodes were placed in the same positions (see Procedure) in the sham and active conditions.

### Procedure

In advance the experimental session, all participants answered the questionnaires and surveys via an online assessment (Unipark^©^). All participants who met all inclusion criteria were invited to the experimental sessions. An experimental session started with seating participants in front of the computer and applying the electrodes. In study 1, the anodal electrode was placed on the scalp over Oz (VC) and the reference electrode was located at Cz (center of the scalp), according to the international 10–20 system of electrode placement. In study 2, the anodal electrode was placed on the scalp over the left dlPPC (F3). Here, the reference electrode was located over the right dlPPC (Fp2).

Afterward, all participants were instructed to perform a short relaxation exercise of 3 min. This exercise focused on breathing and guided attention to several body parts. In order to increase disgust experience, all participants watched a short clip of the film *Trainspotting* (Macdonald and Boyle, 1996) and a short clip of *Pink Flamingo* (Waters, 1972), balanced over the 2 days. Both film clips were approximately 90 s long and have been previously used to induce disgust in experimental settings (e.g., Gross and Levenson, 1995). Hereafter, the instructor used pre-defined codes to either start sham or active tDCS. The use of codes enabled a double-blind study design. The current was increased during the first 5 s of stimulation to 1 mA. In the active conditions, the current was delivered for 20 min (during the whole computer experiment), while in the sham conditions the current was only applied for 40 s.

**FIGURE 1:**
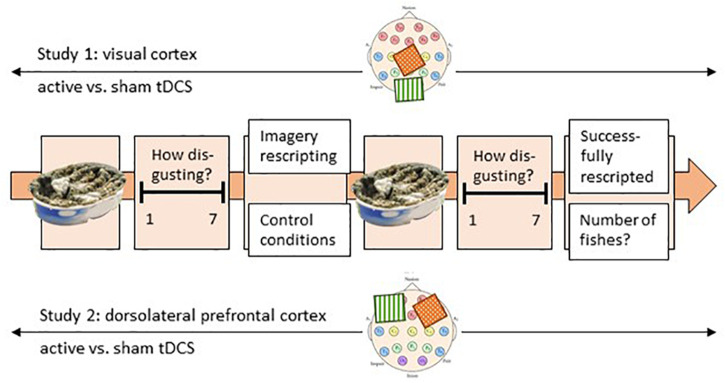
Experimental procedure; presented is one trial. Each participant performed six trials over 2 days. One trial started with the picture presentation for 12 s, and the disgust rating related to this picture (t1). Thereafter, participants performed either the imagery rescripting or the control condition (within-subject emotion-regulation condition). Subsequently, the same picture was presented for 12 s again and rated on a seven-point Likert scale (t2). The experiment was conducted in two groups in two separate but paralleled studies (between-subject). During study 1, the visual cortex and during study 2 the PFC was three times active and three times sham tDC-stimulated. On the two scalp images, the localization of the two electrodes is roughly displayed. The green electrode stands for anodal stimulation, while the red electrode stands for cathodal stimulation (the reference electrode).

After the film clip, participants had to answer fourteen questions (approximately 3–5 min), which were used to select six individually-relevant disgust pictures to be presented in the experiment (see Stimuli and Material). Subsequently, an experimental trial began with instructions to focus on the first picture, which was presented in the center of the screen for 12 s. The participants were asked to indicate on a seven-point Likert scale how disgusting the stimulus in the picture was (t1, *initial disgust experience*). This was followed by one randomly-selected experimental emotion-regulation condition (ImR or control condition, see Experimental Emotion-Regulation Conditions), which lasted approximately 6.5 min. The instructions for ImR were presented audibly and the screen turned white, while in the control conditions the aquarium film was presented without auditory input. A loud noise signaled the end of the intervention, and the same disgust picture was represented for 12 s. It was again followed by the presentation of the seven-point Likert scale and the question about how disgusting the stimulus was (t2). At the end of each experimental trial, the participants were asked to answer detailed questions on what they had actually done during the trial using paper and pencil. In ImR conditions, they were asked whether they had succeeded in rescripting the image, how they had rescripted the image and what name they had chosen for the rescripted image. In the control conditions, participants were instructed to write down the number of times that the yellow fish had appeared. After pressing a key, the next experimental trial started, with a different picture from the same disgust category and one of the two randomly-selected experimental emotion-regulation conditions (Figure 1). Each participant was tested under the two experimental conditions three times each, over 2 days. The experimental conditions were held in a randomized order. In each experimental trial, the same picture was presented before (t1) and after (t2) the experimental emotion-regulation condition. Thus, each participant saw a total of six different pictures over six experimental trials.

### Experimental Emotion-Regulation Conditions

The ImR (Appendix B) condition was used from the experiment of Fink et al. (2018). The participants were instructed to mentally rescript the disgust-inducing picture into a positive picture, in a procedure comprising a series of steps. After the presentation of the auditory instruction, a 10–15 s pause gave the participants the opportunity to follow the instructions. The duration of the procedure was 6.5 min in total. A detailed description and the complete transcription of the instructions can be found in the study of Fink et al. (2018). In the control condition, the participants had to watch a video of an aquarium with moving fishes. They were instructed to count the number of times that a yellow fish swam in and out of the picture. The duration of the procedure was again 6.5 min in total.

### Statistical Analysis

The software R (R Development Core Team, 2012) was used for statistical analysis. ANOVAs and ANCOVAs were calculated with the *ez* R package (Lawrence and Lawrence, 2016) for the dependent variable *difference in disgust experience between t1 and t2*, with negative values indicating disgust reduction and for the dependent variable *initial disgust experience* (t1, before the strategy). The assumption of normality was rejected for both dependent variables, although Vasey and Thayer (1987) showed that an ANOVA with repeated measures is relatively robust to deviations from normality, particularly if other assumptions are not violated (Berkovits et al., 2000). This is the case in the present experiment because sphericity is given (because the repeated measure has only two levels) and there is no relevant drop-out data. Data was aggregated across each of the regulation conditions (ImR or control), each participant and each of the localization conditions (PFC or V1) across trials. The statistical hypotheses were tested at the α = 0.05 (two-tailed) level of significance.

## Results

### The Influence of tDC Stimulation and Localization on Imagery-Based Disgust Reduction (Hypothesis 1a, 1b, and 2)

The *initial disgust experience* (t1, before applying an emotion regulation strategy) was investigated with two 2 × 2 mixed-subject ANOVAs (Appendix C1) for study 1 and study 2, separately. In study 1, there were no significant main effects of emotion regulation *strategy, F*(1,28) = 0.463, *p* = 0.502, η^2^ = 0.016, no significant main effects of *stimulation, F*(1,28) = 0.057, *p* = 0.813, η^2^ = 0.002, and no significant interaction, *F*(1,28) = 0.111, *p* = 0.742, η^2^ = 0.004. In study 2, there were also no significant main effects of *strategy, F*(1,28) = 1.44, *p* = 0.24, η^2^ = 0.049, no significant main effects of *stimulation, F*(1,28) = 0.04, *p* = 0.843, η^2^ = 0.001, and no significant interactions, *F*(1,28) = 0.731, *p* = 0.4, η^2^ = 0.025. Thus, there was no difference in the intensity of the initial disgust experience at the start of each of the conditions.

To test the effect of direct current stimulation and emotion regulation strategy on disgust reduction, two 2 × 2 repeated measure ANOVA (Appendix C1) were conducted with the within-subject factors *stimulation* (active vs. sham) and *strategy* (ImR vs. control conditions) for study 1 (VC) and study 2 (dorsolateral prefrontal cortex), separately. For study 1, there was a significant main effect for *strategy, F*(1,28) = 3.623, *p* = 0.023, η^2^ = 0.172, indicating that ImR, *M* = −.816, *SD* = 1.427, was significantly more successful in reducing disgust compared to the control condition, *M* = −0.425, *SD* = 1.096, *t*(28) = 2.412, *p* = 0.023. There were no significant main effects for *stimulation, F*(1,28) = 0.112, *p* = 0.741, η^2^ = 0.004, and no significant interaction*, F*(1,28) = 0.487, *p* = 0.491, η^2^ = 0.017. For study 2, there was also a significant main effect for *strategy, F*(1,28) = 6.62, *p* = 0.016, η^2^ = 0.191, indicating that ImR, *M* = −0.529, *SD* = 1.055, was significantly more successful in reducing disgust compared to the control condition, *M* = −0.126, *SD* = 1.886, *t*(28) = 2.573, *p* = 0.016. There were no significant main effects for *stimulation, F*(1,28) = 0.615, *p* = 0.439, η^2^ = 0.021, and a marginal significant interaction *strategy* ×*stimulation, F*(1,28) = 3.289, *p* = 0.08, η^2^ = 0.105. However, the disgust reduction during ImR was slightly stronger during sham prefrontal direct current stimulation, *M* = −0.395, *SD* = 0.929, compared to active prefrontal stimulation *M* = −0.659, *SD* = 1.16, a *post hoc* analysis revealed no significant difference, *t*(28) = 1.440, *p* = 0.161, *d* = 0.267.

**FIGURE 2:**
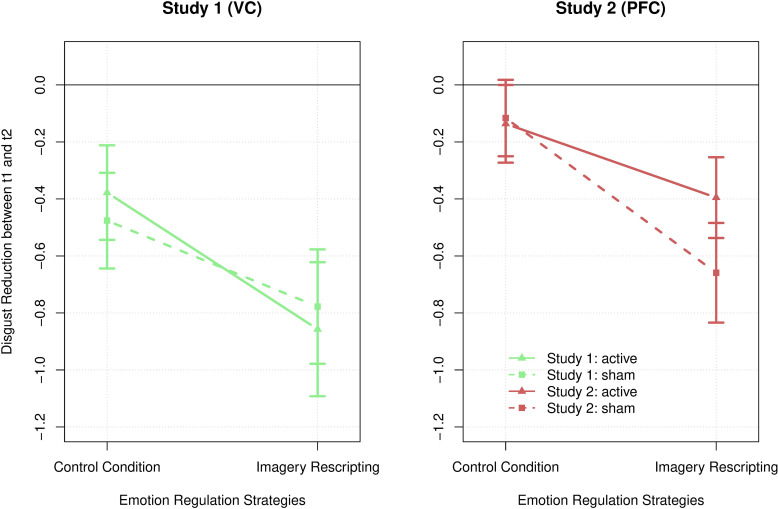
Disgust reduction across both emotion regulation strategies, control condition and imagery rescripting, both *localization* conditions, study 1 on the visual cortex (green) and study 2 on the dorsolateral prefrontal cortex (red), as well as both *stimulation* conditions, active (lines) and sham (dotted lines) tDCS. The error bars represent the standard error.

To answer the explorative hypothesis 2, a 2 × 2 × 2 ANOVA (Appendix C2) with the within-factors *strategy* and *stimulation* and the between-subject factor *group* (or *localization*) was conducted. The results indicate that neither of the two *localization* conditions (visual cortex vs. dorsolateral prefrontal cortex) significantly improved imagery-based disgust reduction. The three-way interaction *strategy × stimulation × localization* became not significant, *F*(1,222) = 2.688, *p* = 0.107, η^2^ = 0.046. There was also no significant interaction of *strategy × stimulation*, *F*(1,56) = 0.268, *p* = 0.607, η^2^ = 0.005, *strategy × localization*, *F*(1,56) = 0.098, *p* = 0.755, η^2^ = 0.001, or *stimulation × localization*, *F*(1,56) = 0.08, *p* = 0.779, η^2^ = 0.001. However, as above, the main effect for *strategy* became significant, *F*(1,56) = 12.429, *p* < 0.001, η^2^ = 0.181. A post-hoc analysis revealed that ImR, *M* = −0.685, *SD* = 1.128, was significantly more successful in reducing disgust compared to the control condition, *M* = −0.297, *SD* = 1.897, *t* = 3.554, *p* < 0.001, *d_cohen_* = 0.467, in both groups. The results are presented in Figure 2.

### The Influence of Personal Traits on Imagery-Based Disgust Reduction (Hypothesis 3a and 3b)

To test the influence of personal traits, ANCOVAs were calculated controlling for the effect of the co-variates *habitual use of spontaneous use of imagery*, *habitual use of cognitive reappraisal*, *habitual use of suppression* and *subclinical contamination-based obsessive–compulsive symptoms* (C-OCS) on the significant main effect *emotion regulation strategy* from the 2 × 2 × 2 ANOVA [see The Influence of tDC Stimulation and Localization on Imagery-Based Disgust Reduction (Hypothesis 1a, 1b, and 2)]. None of the ANCOVA models (Appendix C3) with the dependent variable *difference of disgust experience* between t1 and t2 (disgust reduction), the factor *strategy* and the traits *habitual use of spontaneous use of imagery*, *habitual use of cognitive reappraisal*, *habitual use of suppression* and C-OC symptoms did become significant, *p* > 0.2. This indicates that the traits had no impact on the influence of ImR on disgust reduction. For the ANCOVA with the dependent variable disgust experience at t1 (initial disgust experience), the factor emotion regulation *strategy* was excluded, given that the strategy was applied after the initial disgust experience and should not have any impact. There was a significant effect of *subclinical C-OC symptoms* on initial disgust experience, *F*(1,336) = 15.618, *p* < 0.001, η^2^ = 0.044, indicating that participants with more C-OC symptoms reported a stronger initial disgust experience, *r* = 0.073, *p* = 0.011, *R^2^* = 0.11. All other traits had no impact on the initial disgust experience. The results are listed in Appendix A.

## Discussion

This is the first study to test the effect of transcranial direct current stimulation as an augmentation strategy for reducing disgust via ImR. The study was designed to answer the question of whether tDCS can support the use of ImR on disgust reduction. Therefore, anodal direct current stimulation was applied to two different locations: over the VC and over the dorsolateral prefrontal cortex. The results corroborate the findings of Fink et al. (2018) by showing that disgust reduction was significantly more successful in ImR conditions compared to control conditions. However, tDCS had no significant impact on successful disgust reduction through ImR, either active or sham, whether applied over the VC or over the dorsolateral prefrontal cortex. Based on these results, it is questionable whether tDCS can improve disgust regulation through ImR. Nevertheless, the results contribute to the existing literature by showing how powerful ImR is in reducing disgust and by raising questions of the neural basis of imagery in the context of brain stimulation.

It was particularly surprising that in study 2 active anodal tDCS had no impact on disgust reduction given the widely studied facilitating effect of anodal tDCS over the dlPFC for emotion regulation processes (Tremblay et al., 2014). Therefore, we expected that active anodal tDCS would have resulted in reduced disgust experience during ImR compared to sham stimulation. Instead, the results show a marginally significant sham–active difference, indicating that disgust reduction with ImR (not during control conditions) was particularly strong during sham compared to active prefrontal tDCS. In previous studies, active anodal stimulation of the left dlPFC has been shown to result in decreased negative emotion perception (Boggio et al., 2009) and increased control of negative emotions (Maeoka et al., 2012). It is possible that this leads to the production of an already disgust-reduced mental image, resulting in smaller possible scope for change during ImR. This finding contradicts hypothesis 1b and, although it was only a marginal effect, it raises the question of whether tDCS prefrontal stimulation might even counteract the effect of ImR.

There are studies showing that anodal tDCS over the VC supported visual learning (Sczesny-Kaiser et al., 2016; stimulated with 1 mA) and increased the cortical excitability (Antal et al., 2003). It has been generally suggested that stimulating the VC leads to some area-related improvement. Thus, we expected that stimulating the excitability of the VC would enhance the vividness of the mental image (Cui et al., 2007), especially as there is good evidence that vivid mental imagery is associated with strong emotional experiences (Miller et al., 1987). However, the missing difference of initial disgust experience between sham and active stimulation in study 1 indicates that anodal tDCS over the VC did not result in more vividness of the mental images. Even though the VC plays a crucial role in imagination and perception (Kosslyn et al., 2001) and the prefrontal cortex might play a role in the emotion regulation process underlying ImR, there has been no research so far that has investigated the role of the visual or prefrontal cortex during ImR. If several cortical areas are involved, ImR might not be easily supported by tDCS through stimulation of only one of these regions at a time. This raises the question if, in general, ImR can be supported at all by brain stimulation, and it demonstrates that more research on the cognitive and neural basis of ImR is needed.

In contrast to the missing demonstration of findings for tDCS within the present experimental design, the results show that ImR is a sufficient emotion regulation strategy to change levels of disgust. This is a replication of the findings of Fink et al. (2018). It further supports the application of ImR in changing levels of disgust. The effectiveness of ImR in future experiments might be increased by an even more idiosyncratic procedure. This would include face-to-face instruction, individual disgust stimulus, a longer period of imagination of the stimulus before the intervention and more training sessions. Contrary to hypothesis 3, the habitual use of reappraisal, the habitual use of suppression, the severity of washing symptoms and – most surprisingly – the habitual use of imagery did not have an impact on imagery-based disgust reduction. This suggests that the trait vividness and the accessibility of the mental image did not have an impact on the processing of ImR. This can be also framed in a positive way: it seems that everyone can profit, independently of the investigated predispositions. The only effect found was that people with higher washing symptoms reported stronger initial disgust experience, which indicates that the general disgust induction paradigm worked well.

### Methodological Considerations and Limitations of the Experiment

We wish to highlight the methodological strength of the present study, namely that tDCS was applied to two different locations, using a sham-controlled, double-blinded study. Approved instructions for inducing ImR were applied and compared with control conditions. However, we are also aware of some limitations of our study. First, a non-clinical population limits the external validity concerning clinical implications, although previous research (for reviews, see Abramowitz et al., 2003) has postulated that thoughts and behaviors for those with OCD differ more quantitatively rather than qualitatively than those observed in non-clinical individuals. Furthermore, in the study of Fink et al. (2018), we found no differences in the successful application of ImR for disgust reduction between a C-OCD group and matched healthy controls. The second limitation of this study is the gender ratio of 21 females to 8 males (71%). A more gender-diverse sample might control for disgust-specific gender effects, taking into account the notion that women tend to be more sensitive to disgust (Olatunji et al., 2005). Third, most studies investigating the effect of tDCS in OCD used amperages of 2 mA; therefore, it could be suggested that the use of 1 mA was not sufficient to prompt significant effects of tDCS. On the other hand, other studies have found tDCS effects by only stimulating with 1mA (e.g., Sczesny-Kaiser et al., 2016) and at least tendencies should have been found (they were not) if the results were likely to become significant when applying 2 mA direct current. Furthermore, we recruited healthy participants and because tDCS is not without side effects, we felt the responsibility to find an effective but conservative (not damaging) amount of mA. Fourth, six trials are maybe not enough for a good reliability to show tDCS effects: this is why in future studies more trials are needed. However, because our goal was to show that tDCS improves ImR effects, we would argue that if the effect would have been clinically relevant, at least a tendency should have been found. Fifth, because tDCS was already active during the questionnaire, the selection of the pictures might already have been influenced by tDCS. Because this was similar in both conditions and no pictures were presented, we would argue that the impact of a potential modulating effect on the research question can be considered as small. However, to avoid possible confounding effects, in future studies tDCS should be started after the picture selection process. Sixth, the allocation of the localization conditions was not randomized but parallelized, which should be corrected in future studies. Finally, in future studies an auditory or imaginary control condition should be used for better comparability.

## Conclusion and Implications for Future Research

The results corroborate the findings of the study of Fink et al. (2018), showing that ImR seems to be a successful disgust regulation strategy. This finding should encourage researchers to further investigate ImR for disgust regulation in more elaborate settings (longer durations, idiosyncratic disgust images, face-to-face interactions) and should further encourage therapists to test ImR in therapeutic settings in addition to ERP. The results found that with this experimental design no impact of active tDCS could be found, neither when applied over the VC or over the dorsolateral cortex. The findings of the present study raise questions as to the neural basis of ImR: if ImR can be supported through brain stimulation at all, and more specifically, if prefrontal stimulation might even reduce the effect of ImR on disgust. Therefore, further fMRI studies should investigate the neural basis of ImR before further investigating brain stimulation in this context. With our theoretically derived localization positions, we were not able to show effects of tDCS on ImR with this experimental design.

## Data Availability

The raw data supporting the conclusions of this manuscript will be made available by the authors, without undue reservation, to any qualified researcher.

## Ethics Statement

This study was carried out in accordance with the recommendations of the local ethics committee of the University of Leipzig with written informed consent from all subjects. All subjects gave written informed consent in accordance with the Declaration of Helsinki. The protocol was approved by the local ethics committee of the University of Leipzig (458-15-21122015).

## Author Contributions

JF developed the research idea, conducted the data collection, evaluated the data, and wrote the rough draft of the manuscript. CE developed the research idea, supervised the research project, supported the data evaluation, and contributed substantially to the drafting of the manuscript.

## Conflict of Interest Statement

The authors declare that the research was conducted in the absence of any commercial or financial relationships that could be construed as a potential conflict of interest.

## Footnotes

1 An extra survey was conducted to measure tiredness, mood, medication, drug use, alcohol use, nicotine use, hunger, coffee consumption, attentional focus and pain experience; furthermore, the survey assessed general discomfort over the last 48 h. Additionally, another survey measured experience with progressive muscle relaxation, autogenic training, hypnosis, mindfulness-based strategies, meditation, yoga and relaxation in movement. However, none of these variables had theoretical implications for the aim of the present study and were therefore not analyzed.

2 The Disgust-RelaTed-Images database and technical manuals (Haberkamp et al., 2017) can be obtained on request from the original authors. DIRTI numbers for disgust pictures used in this experiment are 1034, 1038, 1051, 1072, 1073, 1116, 1118, 1119, 1134, 1135, 1139, 1209, 1225, 1238, 1253, 1258.

## References

[B1] Abler B. Kessler H. (2009). Emotion regulation questionnaire – eine deutschsprachige fassung des ERQ von Gross und John. *Diagnostica* 55 144–152. 10.1026/0012-1924.55.3.144

[B2] Abramowitz J. S. Franklin M. E. Schwartz S. A. Furr J. M. (2003). Symptom presentation and outcome of cognitive-behavioral therapy for obsessive-compulsive disorder. *J. Consult. Clin. Psychol.* 71 1049–1057. 10.1037/0022-006X.71.6.1049 14622080

[B3] Ackenheil M. Stotz G. Dietz-Bauer R. Vossen A. (1999). *Deutsche Fassung des Mini-International Neuropsychiatric Interview.* München: Psychiatrische Universitätsklinik München.

[B4] Adams T. G. Willems J. L. Bridges A. J. (2011). Contamination aversion and repeated exposure to disgusting stimuli. *Anxiety Stress Coping* 24 157–165. 10.1080/10615806.2010.506953 20737324

[B5] Antal A. Kincses T. Z. Nitsche M. A. Paulus W. (2003). Manipulation of phosphene thresholds by transcranial direct current stimulation in man. *Exp. Brain Res.* 150 375–378. 10.1007/s00221-003-1459-8 12698316

[B6] Bais M. Figee M. Denys D. (2014). Neuromodulation in obsessive-compulsive disorder. *Psychiatr. Clin. North Am.* 37 393–413. 10.1016/j.psc.2014.06.003 25150569

[B7] Berkovits I. Hancock G. R. Nevitt J. (2000). Bootstrap resampling approaches for repeated measure designs: relative robustness to sphericity and normality violations. *Educ. Psychol. Meas.* 60 877–892. 10.1177/00131640021970961

[B8] Boggio P. S. Zaghi S. Fregni F. (2009). Modulation of emotions associated with images of human pain using anodal transcranial direct current stimulation (tDCS). *Neuropsychologia* 47 212–217. 10.1016/j.neuropsychologia.2008.07.022 18725237

[B9] Brunelin J. Mondino M. Bation R. Palm U. Saoud M. Poulet E. (2018). Transcranial direct current stimulation for obsessive-compulsive disorder: a systematic re-view. *Brain Sci.* 8:E37. 10.3390/brainsci8020037 29495298 PMC5836056

[B10] Burns G. L. Keortge S. G. Formea G. M. Sternberger L. G. (1996). Revision of the Padua inventory of obsessive compulsive disorder symptoms: distinctions between worry, obsessions, and compulsions. *Behav. Res. Ther.* 34 163–173. 10.1016/0005-7967(95)00035-6 8741724

[B11] Cisler J. M. Brady R. E. Olatunji B. O. Lohr J. M. (2010). Disgust and obsessive beliefs in contamination-related OCD. *Cogn. Ther. Res.* 34 439–448. 10.1007/s10608-009-9253-y 20877585 PMC2945391

[B12] Cougle J. R. Wolitzky-Taylor K. B. Lee H.-J. Telch M. J. (2007). Mechanisms of change in ERP treatment of compulsive hand washing: does primary threat make a difference? *Behav. Res. Ther.* 45 1449–1459. 10.1016/j.brat.2006.12.001 17240352

[B13] Cui X. Jeter C. B. Yang D. Montague P. R. Eagleman D. M. (2007). Vividness of mental imagery: individual variability can be measured objectively. *Vis. Res.* 47 474–478. 10.1016/j.visres.2006.11.013 17239915 PMC1839967

[B14] Davis N. J. (2014). Transcranial stimulation of the developing brain: a plea for extreme caution. *Front. Hum. Neurosci.* 8:137. 10.3389/fnhum.2014.00600 25140146 PMC4122183

[B15] Faul F. Erdfelder E. Lang A.-G. Buchner A. (2007). G^∗^Power 3: a flexible statistical power analysis program for the social, behavioral, and biomedical sciences. *Behav. Res. Methods* 39 175–191. 10.3758/BF03193146 17695343

[B16] Feeser M. Prehn K. Kazzer P. Mungee A. Bajbouj M. (2014). Transcranial direct current stimulation enhances cognitive control during emotion regulation. *Brain Stimul.* 7 105–112. 10.1016/j.brs.2013.08.006 24095257

[B17] Fink J. Pflugradt E. Stierle C. Exner C. (2018). Changing disgust through imagery rescripting and cognitive reappraisal in contamination-based obsessive-compulsive disorder. *J. Anxiety Disord.* 54 36–48. 10.1016/j.janxdis.2018.01.002 29421371

[B18] Gönner S. Ecker W. Leonhart R. Limbacher K. (2010). Multidimensional assessment of OCD: integration and revision of the Vancouver Obsessional-compulsive inventory and the symmetry ordering and arranging questionnaire. *J. Clin. Psychol.* 66 739–757. 10.1002/jclp.20690 20527054

[B19] Görgen S. M. Hiller W. Witthöft M. (2016). Die spontaneous use of imagery scale (SUIS)–Entwicklung und teststatistische Prüfung einer deutschen adaption. *Diagnostica* 62 31–43. 10.1026/0012-1924/a000135

[B20] Gross J. J. John O. P. (2003). Individual differences in two emotion regulation processes: implications for affect, relationships, and well-being. *J. Pers. Soc. Psychol.* 85 348–362. 10.1037/0022-3514.85.2.348 12916575

[B21] Gross J. J. Levenson R. W. (1995). Emotion elicitation using films. *Cogn. Emot.* 9 87–108. 10.1080/02699939508408966

[B22] Haberkamp A. Glombiewski J. A. Schmidt F. Barke A. (2017). The disgust-related-images (DIRTI) database: validation of a novel standardized set of disgust pictures. *Behav. Res. Ther.* 89 86–94. 10.1016/j.brat.2016.11.010 27914317

[B23] Holmes E. A. Mathews A. (2005). Mental imagery and emotion: a special relationship? *Emotion* 5 489–497. 10.1037/1528-3542.5.4.489 16366752

[B24] Kleiner M. Brainard D. H. Pelli D. G. (2007). What’s new in Psychtoolbox-3. *Perception* 36 1–16.

[B25] Kosslyn S. M. Ganis G. Thompson W. L. (2001). Neural foundations of imagery. *Nat. Rev. Neurosci.* 2 635–642. 10.1038/35090055 11533731

[B26] Lawrence M. A. Lawrence M. M. A. (2016). *ez: Easy Analysis and Visualization of Factorial Experiments. R-Package: Version 4.4-0.* Available at: http://github.com/mike-lawrence/ez

[B27] Lecrubier Y. Sheehan D. V. Weiller E. Amorim P. Bonora I. Harnett Sheehan K. (1997). The mini international neuropsychiatric interview (MINI). A short diagnostic structured interview: reliability and validity according to the CIDI. *Eur. Psychiatry* 12 224–231. 10.1016/S0924-9338(97)83296-8

[B28] Macdonald A. Boyle D. (1996). *Trainspotting [Film].* York: York press.

[B29] Maeoka H. Matsuo A. Hiyamizu M. Morioka S. Ando H. (2012). Influence of transcranial direct current stimulation of the dorsolateral prefrontal cortex on pain related emotions: a study using electroencephalographic power spectrum analysis. *Neurosci. Lett.* 512 12–16. 10.1016/j.neulet.2012.01.037 22326385

[B30] Mason E. C. Richardson R. (2010). Looking beyond fear: the extinction of other emotions implicated in anxiety disorders. *J. Anxiety Disord.* 24 63–70. 10.1016/j.janxdis.2009.08.007 19747796

[B31] Miller G. A. Levin D. N. Kozak M. J. Cook E. W. McLean A. Lang P. J. (1987). Individual differences in imagery and the psychophysiology of emotion. *Cogn. Emot.* 1 367–390. 10.1080/02699938708408058

[B32] Morina N. Lancee J. Arntz A. (2017). Imagery rescripting as a clinical intervention for aversive memories: a meta-analysis. *J. Behav. Ther. Exp. Psychiatry* 55 6–15. 10.1016/j.jbtep.2016.11.003 27855298

[B33] Nelis S. Holmes E. A. Griffith J. W. Raes F. (2014). Mental imagery during daily life: psychometric evaluation of the spontaneous use of Im-agery Scale (SUIS). *Psychol. Belg.* 54 19–32. 10.5334/pb.ag 26290615 PMC4538780

[B34] Nitsche M. A. Paulus W. (2000). Excitability changes induced in the human motor cortex by weak transcranial direct current stimulation. *J. Physiol.* 527 633–639. 10.1111/j.1469-7793.2000.t01-1-00633.x 10990547 PMC2270099

[B35] Olatunji B. O. Sawchuk C. N. Arrindell W. A. Lohr J. M. (2005). Disgust sensitivity as a mediator of the sex differences in contamination fears. *Pers. Individ. Differ.* 38 713–722. 10.1016/j.paid.2004.05.025

[B36] Olatunji B. O. Wolitzky-Taylor K. B. Willems J. L. Lohr J. M. Armstrong T. (2009). Differential habituation of fear and disgust during repeated exposure to threat-relevant stimuli in contamination-based OCD: an analogue study. *J. Anxiety Disord.* 23 118–123. 10.1016/j.janxdis.2008.04.006 18541403

[B37] Pearson J. Naselaris T. Holmes E. A. Kosslyn S. M. (2015). Mental imagery: functional mechanisms and clinical applications. *Trends Cogn. Sci.* 19 590–602. 10.1016/j.tics.2015.08.003 26412097 PMC4595480

[B38] Peña-Gómez C. Vidal-Piñeiro D. Clemente I. C. Pascual-Leone,Á Bartr és-Faz D. Aleman A. (2011). Down-regulation of negative emotional processing by transcranial direct current stimula-tion: effects of personality characteristics. *PLoS One* 6:e22812. 10.1371/journal.pone.0022812 21829522 PMC3146508

[B39] R Development Core Team (2012). *R: A Language and Environment for Statistical Computing.* Vienna: R foundation for statistical computing.

[B40] Reisberg D. Pearson D. G. Kosslyn S. M. (2003). Intuitions and introspections about imagery: the role of imagery experience in shaping an investigator’s theoretical views. *Appl. Cognit. Psychol.* 17 147–160. 10.1002/acp.858

[B41] Rosa-Alcazar A. I. Sanchez-Meca J. Gomez-Conesa A. Marin-Martinez F. (2008). Psychological treatment of obsessive–compulsive disorder: a meta-analysis. *Clin. Psychol. Rev.* 28 1310–1325. 10.1016/j.cpr.2008.07.001 18701199

[B42] Sczesny-Kaiser M. Beckhaus K. Dinse H. R. Schwenkreis P. Tegenthoff M. Höffken O. (2016). Repetitive transcranial direct current stimulation induced excitability changes of primary visual cortex and visual learning effects-a pilot study. *Front. Behav. Neurosci.* 10:261. 10.3389/fnbeh.2016.00116 27375452 PMC4891342

[B43] Smits J. Telch M. Randall P. (2002). An examination of the decline in fear and disgust during exposure-based treatment. *Behav. Res. Ther.* 40 1243–1253. 10.1016/S0005-7967(01)00094-8 12384321

[B44] Tremblay S. Lepage J.-F. Latulipe-Loiselle A. Fregni F. Pascual-Leone Á Théoret H. (2014). The uncertain outcome of prefrontal tDCS. *Brain Stimul.* 7 773–783. 10.1016/j.brs.2014.10.003 25456566 PMC4342747

[B45] Vasey M. W. Thayer J. F. (1987). The continuing problem of false positives in repeated measures ANOVA in psychophysiology: a multivariate solution. *Psychophysiology* 24 479–486. 10.1111/j.1469-8986.1987.tb00324.x 3615759

[B46] Waters J. (1972). *Pink Flamingos [Film].* Maryland, MD: Dreamland.

[B47] Wild J. Hackmann A. Clark D. M. (2008). Rescripting early memories linked to negative images rescripting early memories linked to negative images in social phobia: a pilot study. *Behav Ther* 39 47–56. 10.1016/j.beth.2007.04.003 18328869 PMC2270350

