# Corrigendum: Does Transcranial Direct Current Stimulation (tDCS) Improve Disgust Regulation Through Imagery Rescripting?

**DOI:** 10.3389/fnhum.2020.00263

**Published:** 2020-07-31

**Authors:** Jakob Fink, Cornelia Exner

**Affiliations:** Department of Clinical Psychology and Psychotherapy, University of Leipzig, Leipzig, Germany

**Keywords:** transcranial direct current stimulation, brain stimulation, disgust, imagery rescripting, emotion regulation, visual cortex, prefrontal cortex, neuropsychological mechanism

In the original article, there was an error. In the following sentence on page 4 the numbers concerning the international 10-20 system of electrode placement were interchanged. There it says: “*In study 2, the anodal electrode was placed on the scalp over the left dlPPC (F2). Here, the reference electrode was located over the right dlPPC (Fp3).”*

A correction has been made to *Materials and Methods, Procedure, first paragraph:*

“In study 2, the anodal electrode was placed on the scalp over the left dlPPC (F3). Here, the reference electrode was located over the right dlPPC (Fp2).”

The authors apologize for this error and state that this does not change the scientific conclusions of the article in any way. The original article has been updated.

