# Supplementary material for: Does Transcranial Direct Current Stimulation (tDCS) Improve Disgust Regulation Through Imagery Rescripting?
Source: Front Hum Neurosci. 2019 Jun 6;13:192. doi: 10.3389/fnhum.2019.00192 (PMC6593270; doi:10.3389/fnhum.2019.00192)
Supplement: Supplementary file 1 [file Data_Sheet_1.docx]

Appendix A

Table. ANCOVAs model fit parameters.

| Model | | *df* | *α* | *β* | *x* | *β*x* | *R²* |
| --- | --- | --- | --- | --- | --- | --- | --- |
|  | *Dependent variable: Difference in disgust experience between t1 and t2* | | | | | | |
| strategy * SUIS | | 3,112 | -.537 | -.626 | .004 | .004 | .063 |
| strategy * reappraisal | | 3,112 | -.463 | -.122 | .039 | -.056 | .058 |
| strategy * suppression | | 3,112 | -.034 | -.282 | -.081 | -.038 | .070 |
| strategy * PI washing | | 3,112 | -.321 | -.245 | .007 | -.024 | .063 |
|  | *Dependent variable: Initial disgust experience t1* | | | | | | |
| SUIS | | 1,56 | 3.311*** | - | .003 | - | <.001 |
| reappraisal | | 1,56 | 4.124*** | - | -.129 | - | .008 |
| suppression | | 1,56 | 3.670*** | - | -.058 | - | .003 |
| PI washing | | 1,56 | 3.027*** | - | .073* | - | .110 |

SUIS = Spontaneous Use of Imagery Scale, PI washing = Padua Inventory – washing subscale, reappraisal and suppression were calculated with the Emotion Regulation Questionnaire (ERQ), α = model intercept, β = effect of emotion regulation strategy, x = effect of traits, β*x = interaction, R² = multiple R squared, * = *p* < .05, *** = *p* < .001.

# Appendix B: Imagery Rescripting Procedure

| **Instructions** | **Aspirated functions** | **time** |
| --- | --- | --- |
| Envision the picture before your inner eye.  To do so, you can either close your eyes or look at the screen.  Imagine as detailed as possible what you can see, listen, feel and smell. | Introduction, activation of experiencing | 0:00-0:31 |
| Imagine how you enter the picture and how the presented object becomes real.  Your body is surrounded by a protective cover. Nothing can penetrade the cover to threaten you.  Imagine your protection cover as detailed as possible. What color does it have? How big is it? | Activation of imagery and trial imagery as a tool. Protective cover as protection of threat; increases feelings of control | 0:31-1:25 |
| Please move yourself inside the picture towards the presented object.  Use your fantasy to change the size of the presented object.  At first, make the object very tiny.  Afterwards make it huge.  Return it to the normal size. | Activation of imagery and working with imagery as a tool. | 1:25-2:03 |
| Please notice exactly what the object looks like, how the object sounds and how the object smells. | Activation of experiencing and feeling of disgust | 2:03-2:28 |
| If you are disgusted, try to tolerate this feeling | Activation of experiencing and feeling of disgust | 2:28-2:40 |
| Please try to change the object in a way that it is not disgusting anymore.  In your imagination you can be a wizard. You can change everything! You decide, what happens in your imagination.  You can include new elements and you can erase elements in your imagination.  You can change the environment or you can change the object into something completely different.  There are no limits. Let your imagination go wild! | Imagery Rescripting; testing different options in handling imagery rescripting; increase feelings of control concerning mental experiences | 2:40-4:09 |
| Develop your own positive image of the object!  What do you observe in your changed image?  Move through your image. Are you satisfied with the changes?  You can change the scenery as long as it takes until you feel good and safe in your image. | Set a criterion to finish imagery rescripting (positive, satisfaction, safety and well-being); activation of experiencing the positive image | 4:09-5:06 |
| Imagine exactly what you see, hear, feel and smell when you look at your new image. | activation of experiencing the positive image | 5:06-5:26 |
| Consider a title or name for your self-created, positive image! | Set a memory-link for fast recall | 5:26-5:41 |
| Please take your attention back to the room. Feel the seat and the position of your arms and legs.  If you want, you can now move and open your eyes. | Return into the present | 5:41-6:30 |

| Appendix C1. ANOVAs to the dependent variables disgust experience at t1 and disgust reduction between t1 and t2 | | | | | | | |
| --- | --- | --- | --- | --- | --- | --- | --- |
|  | | *Sum of Squares* | *df* | *Mean Square* | *F* | *p* | η² |
| ***Disgust experience t1*** | |  |  |  |  |  |  |
| **V3 (study 1)** | |  |  |  |  |  |  |
|  | *stimulation* | .175 | 1 | .175 | .057 | .813 | .002 |
|  | *Residual* | 86.138 | 28 | 3.076 |  |  |  |
|  | *strategy* | .623 | 1 | .623 | .463 | .502 | .016 |
|  | *Residual* | 37.690 | 28 | 1.346 |  |  |  |
|  | *strategy ✻ stimulation* | .106 | 1 | .106 | .111 | .742 | .004 |
|  | *Residual* | 26.707 | 28 | .954 |  |  |  |
| **PFC (study 2)** | |  |  |  |  |  |  |
|  | *stimulation* | .106 | 1 | .106 | .040 | .843 | .001 |
|  | *Residual* | 74.207 | 28 | 2.650 |  |  |  |
|  | *strategy* | 1.140 | 1 | 1.140 | 1.440 | .240 | .049 |
|  | *Residual* | 22.172 | 28 | .792 |  |  |  |
|  | *strategy ✻ stimulation* | .364 | 1 | .364 | .731 | .400 | .025 |
|  | *Residual* | 13.948 | 28 | .498 |  |  |  |
| ***Disgust reduction between t1 and t2*** | |  |  |  |  |  |  |
| **V3 (study 1)** | |  |  |  |  |  |  |
|  | *stimulation* | .106 | 1 | .106 | .112 | .741 | .004 |
|  | *Residual* | 26.457 | 28 | .945 |  |  |  |
|  | *strategy* | 3.623 | 1 | 3.623 | 5.817 | .023 | .172 |
|  | *Residual* | 17.440 | 28 | .623 |  |  |  |
|  | *strategy ✻ stimulation* | .364 | 1 | .364 | .487 | .491 | .017 |
|  | *Residual* | 20.948 | 28 | .748 |  |  |  |
| **PFC (study 2)** | |  |  |  |  |  |  |
|  | *stimulation* | .485 | 1 | .485 | .615 | .435 | .021 |
|  | *Residual* | 22.078 | 28 | .788 |  |  |  |
|  | *strategy* | 5.175 | 1 | 5.175 | 6.620 | .016 | .191 |
|  | *Residual* | 21.888 | 28 | .782 |  |  |  |
|  | *strategy ✻ stimulation* | 1.347 | 1 | 1.347 | 3.289 | .08 | .105 |
|  | *Residual* | 11.466 | 28 | .409 |  |  |  |
|  | | | | | | |  |
| *Note.*  Type III Sum of Squares | | | | | | |  |

| Appendix C2. 2x2x2 ANOVA to the dependent variable disgust reduction between t1 and t2 | | | | | |  |
| --- | --- | --- | --- | --- | --- | --- |
|  | *Sum of Squares* | *df* | *Mean Square* | *F* | *p* | η² |
| **Within Subjects Effetcs** |  |  |  |  |  |  |
| *stimulation* | .522 | 1 | .522 | .602 | .441 | .011 |
| *stimulation ✻ localization* | .069 | 1 | .069 | .080 | .779 | .001 |
| *Residual* | 48.534 | 56 | .867 |  |  |  |
| *strategy* | 8.728 | 1 | 8.728 | 12.429 | < .001 | ,181 |
| *strategy ✻ localization* | .069 | 1 | .069 | .098 | .755 | .001 |
| *Residual* | 39.328 | 56 | .702 |  |  |  |
| *stimulation ✻ strategy* | .155 | 1 | .155 | .268 | .607 | .005 |
| *stimulation ✻ strategy ✻ localization* | 1.556 | 1 | 1.556 | 2.688 | .107 | .046 |
| *Residual* | 32.414 | 56 | .579 |  |  |  |
|  |  |  |  |  |  |  |
| **Between Subjects Effects** |  |  |  |  |  |  |
| *localization* | 6.556 | 1 | 6.556 | 3.351 | .072 | .056 |
| *Residual* | 109.552 | 56 | 1.956 |  |  |  |
|  | | | | | |  |
| *Note.*  Type III Sum of Squares | | | | | |  |

| **Appendix C3. ANCOVAs** | | | | | | | | | | | | | | |
| --- | --- | --- | --- | --- | --- | --- | --- | --- | --- | --- | --- | --- | --- | --- |
| **Cases** |  | **Sum of Squares** |  | **df** |  | **Mean Square** |  | **F** |  | **p** |  | **η²** |  |  |
| **Difference between t1 and t2** |  |  |  |  |  |  |  |  |  |  |  |  |  |  |
| ERCond |  | 13.612 |  | 1 |  | 13.612 |  | 10.597 |  | .001 |  | .029 |  |  |
| Exp |  | 10.433 |  | 1 |  | 10.433 |  | 8.122 |  | .005 |  | .022 |  |  |
| code |  | .375 |  | 1 |  | .375 |  | .292 |  | .589 |  | .001 |  |  |
| ERQ_Reappraisal |  | .227 |  | 1 |  | .227 |  | .177 |  | .675 |  | <.001 |  |  |
| ERQ_Unterdr.cken |  | 4.412 |  | 1 |  | 4.412 |  | 3.435 |  | .065 |  | .009 |  |  |
| SUIS16 |  | 3.209 |  | 1 |  | 3.209 |  | 2.498 |  | .115 |  | .007 |  |  |
| PIWash |  | 1.438 |  | 1 |  | 1.438 |  | 1.119 |  | .291 |  | .003 |  |  |
| ERCond ✻ Exp |  | .002 |  | 1 |  | .002 |  | .002 |  | .966 |  | <.001 |  |  |
| ERCond ✻ code |  | .030 |  | 1 |  | .030 |  | .023 |  | .878 |  | <.001 |  |  |
| Exp ✻ code |  | .274 |  | 1 |  | .274 |  | .213 |  | .644 |  | .001 |  |  |
| ERCond ✻ Exp ✻ code |  | 1.225 |  | 1 |  | 1.225 |  | .954 |  | .329 |  | .003 |  |  |
| Residual |  | 431.604 |  | 336 |  | 1.285 |  |  |  |  |  |  |  |  |
| **Disgust experience at t1** |  |  |  |  |  |  |  |  |  |  |  |  |  |  |
| ERCond ✻ Exp |  | 3.292 |  | 1 |  | 3.292 |  | 1.217 |  | .271 |  | .003 |  |  |
| code ✻ Exp |  | 0 |  | 0 |  |  |  |  |  |  |  | 0 |  |  |
| ERCond ✻ code ✻ Exp |  | 0 |  | 0 |  |  |  |  |  |  |  | 0 |  |  |
| ERQ_Reappraisal |  | 3.092 |  | 1 |  | 3.092 |  | 1.143 |  | .286 |  | .003 |  |  |
| ERQ_Suppression |  | 1.083 |  | 1 |  | 1.083 |  | .400 |  | .527 |  | .001 |  |  |
| SUIS16 |  | .152 |  | 1 |  | .152 |  | .056 |  | .813 |  | <.001 |  |  |
| **PIWash** |  | **42.254** |  | **1** |  | **42.254** |  | **15.618** |  | **< .001***** |  | **.044** |  |  |
| ERCond |  | .010 |  | 1 |  | .010 |  | .004 |  | .953 |  | <.001 |  |  |
| code |  | .591 |  | 1 |  | .591 |  | .218 |  | .640 |  | .001 |  |  |
| Exp |  | 1.141 |  | 1 |  | 1.141 |  | .422 |  | .516 |  | .001 |  |  |
| ERCond ✻ code |  | .185 |  | 1 |  | .185 |  | .068 |  | .794 |  | <.001 |  |  |
| Residual |  | 909.028 |  | 336 |  | 2.705 |  |  |  |  |  |  |  |  |
|  |  |  |  |  |  |  |  |  |  |  |  |  |  |  |
|  | | | | | | | | | | | | | | |

**Appendix D:**  **Examplified balancing of the emotion regulation conditions, the TDCS conditions and the localization across the participants**

| Participant | day | Block | Trial | Emotion Regulation | TDCS | Localization |
| --- | --- | --- | --- | --- | --- | --- |
| 1 | 1 | 1 | 1 | Imagery | Active | VC |
| 1 | 1 | 2 | 2 | Control | Active | VC |
| 1 | 1 | 3 | 3 | Imagery | Active | VC |
| 1 | 2 | 1 | 4 | Control | Sham | VC |
| 1 | 2 | 2 | 5 | Imagery | Sham | VC |
| 1 | 2 | 3 | 6 | Control | Sham | VC |
| 2 | 1 | 1 | 1 | Control | Active | VC |
| 2 | 1 | 2 | 2 | Imagery | Active | VC |
| 2 | 1 | 3 | 3 | Control | Active | VC |
| 2 | 2 | 1 | 4 | Imagery | Sham | VC |
| 2 | 2 | 2 | 5 | Control | Sham | VC |
| 2 | 2 | 3 | 6 | Imagery | Sham | VC |
| … |  |  |  |  |  |  |
| 30 | 1 | 1 | 1 | Control | Sham | PFC |
| 30 | 1 | 2 | 2 | Imagery | Sham | PFC |
| 30 | 1 | 3 | 3 | Control | Sham | PFC |
| 30 | 2 | 1 | 4 | Imagery | Active | PFC |
| 30 | 2 | 2 | 5 | Control | Active | PFC |
| 30 | 2 | 3 | 6 | Imagery | Active | PFC |
| 31 | 1 | 1 | 1 | Control | Active | PFC |
| 31 | 1 | 2 | 2 | Imagery | Active | PFC |
| 31 | 1 | 3 | 3 | Control | Active | PFC |
| 31 | 2 | 1 | 4 | Imagery | Sham | PFC |
| 31 | 2 | 2 | 5 | Control | Sham | PFC |
| 31 | 2 | 3 | 6 | Imagery | Sham | PFC |
| … |  |  |  |  |  |  |
